# Repositioning of the antipsychotic drug TFP for sepsis treatment

**DOI:** 10.1007/s00109-019-01762-4

**Published:** 2019-03-08

**Authors:** Jung Hwa Park, Hyun Jin Park, Sung Eun Lee, Young Seob Kim, Gun-Young Jang, Hee Dong Han, In Duk Jung, Kyung Chul Shin, Young Min Bae, Tae Heung Kang, Yeong-Min Park

**Affiliations:** 1 Department of Immunology, School of Medicine, KonKuk University, 268, Chungwondaero, Chungju, 27478 South Korea; 2 Department of Physiology, School of Medicine, KonKuk University, Chungju, 27478 South Korea

**Keywords:** Sepsis, Cytokine, TFP, Calmodulin (CaM), Inflammation

## Abstract

**Abstract:**

Sepsis is a disease responsible for the death of almost all critical patients. Once infected by virus or bacteria, patients can die due to systemic inflammation within a short period of time. Cytokine storm plays an essential role in causing organ dysfunction and septic shock. Thus, inhibition of cytokine secretion is considered very important in sepsis therapy. In this study, we found that TFP, an antipsychotic drug mainly used to treat schizophrenia by suppressing dopamine secretion, inhibited cytokine release from activated immune cells both in vitro and in vivo. Trifluoperazine (TFP) decreased the levels of pro-inflammatory cytokines without altering their transcription level. In LPS-induced endotoxemia and cecal content injection (CCI) models, TFP intraperitoneal administration improved survival rate. Thus, TFP was considered to inhibit the secretion of proteins through a mechanism similar to that of W7, a calmodulin inhibitor. Finally, we confirmed that TFP treatment relieved organ damage by estimating the concentrations of aspartate transaminase (AST), alanine transaminase (ALT), and blood urea nitrogen (BUN) in the serum. Our findings were regarded as a new discovery of the function of TFP in treating sepsis patients.

**Key messages:**

• TFP inhibits LPS-induced activation of DCs by suppressing pro-inflammatory cytokine.

• Treatment of TFP increases survival of LPS-induced endotoxemia and CCI sepsis models.

• TFP exerted a protective effect against tissue or organ damage in animal models.

**Electronic supplementary material:**

The online version of this article (10.1007/s00109-019-01762-4) contains supplementary material, which is available to authorized users.

## Introduction

Sepsis, which is caused by a various bacterial and viral infections, leads to a systemic activation of the innate immune system. As a result of the inflammatory response induced by infected cells, phagocytic cells are activated and release pro-inflammatory cytokine [1]. During infection, dendritic cells (DCs) play an essential interface between innate and adaptive immunity and exist in most tissues of the reticuloendothelial system, including all lymphoid organs. They are especially prominent in tissues that are exposed to the external environment where frequent exposure to foreign antigen and microorganisms occurs [2]. Therefore, at the early stage of inflammation, the activated DCs induce immunosuppression reducing pro-inflammatory cytokine production [3]. In sepsis, since the release of the initial inflammatory cytokine causes cytokine storm and eventually results in death due to loss of organ functions such as lung injection, recent studies emphasized the importance of DC, which improve the aberrant immune response and prolong the life during sepsis progression, in the therapeutic strategy target [4, 5]. To date, a large number of therapeutic agents have been developed to treat sepsis, such as antibodies against lipopolysaccharides (LPS), toll-like receptor 4 (TLR4) agonists, antitumor necrosis factor (TNF) agents, drugs targeting platelet-activating factor (PAF), and drugs targeting coagulation cascades [6–8]. In a clinical trial, Xigris, which is a recombinant human form of protein C, blocks coagulation, inhibits inflammatory effects, and preserves organ function [9–11]. However, the result was unsuccessful owing to side effects, and the drug was withdrawn from the market in 2012. The specific drug was not yet approved for clinical use to treat sepsis. Once pathogenic organisms invade a host, they spread through the blood stream. In response to systemic infectious agents, innate immune cells are activated and cause cytokine emission. The rapidly increasing level of pro-inflammatory cytokines causes cytokine storm in the host [12], inducing organ injury or tissue damage, and this phenomenon is defined as a severe sepsis. Finally, septic shock, which is abnormal distribution of blood flow, results in inadequate blood supply to the body tissues, causing ischemia and organ dysfunction and leading to the death of patients [13]. In general, disrupting cytokine storm is very important in determining the mortality of sepsis patients [14]. Thus, the regulation of cytokine secretion should be studied and considered a key role in sepsis treatment.

Trifluoperazine (TFP) is a clinical antipsychotic drug approved in 2001 and primarily used to treat schizophrenia caused by excess of dopamine [15]. TFP exerts an antipsychotic effect by blocking central dopamine and serotonin receptor in patients suffering from megalomania and hallucinations [16]. This agent is also known to function as a calmodulin inhibitor, which prevents calcium from binding to calmodulin (CaM), thereby leading to the elevation of cytosolic calcium level. In other words, the mode of action of TFP is binding to a well-known Ca^2+^-binding protein, “calmodulin.” It has been reported that TFP exerts an inhibitory effect on the function of CaM by directly binding to CaM [17] . CaM is a multifunctional Ca^2+^-binding protein and involved in the activity of various target proteins, such as kinase and phosphatases, to regulate cellular processes, including cell proliferation, development, and secretion [18, 19]. Consequently, calmodulin has been reported to potentially play a role in the secretion of thyroid hormone from the thyroid [20]. Thus, calcium balance and homeostasis is important in protein secretion [21] . According to a previous study, LPS-induced TNF-α production is inhibited by Ca^2+^ chelation and CaM inhibition, and elevating macrophage intracellular Ca^2+^ augments pro-inflammatory cytokine production [22, 23]. In addition, in aberrant inflammation such as sepsis, disrupted calcium homeostasis is known to exacerbate organ dysfunction and accelerate septic shock [24, 25].

In this study, we identified that TFP as a calmodulin inhibitor reduced inflammatory response by suppressing cytokine secretion in LPS-stimulated macrophages and dendritic cells. Ultimately, TFP administration increased the survival rate of LPS-induced endotoxemia model and cecal content injection (CCI) model by preventing cytokine secretion in the serum and normalizing pathogen infection-induced tissue damage and organ dysfunction. These findings suggested that TFP as a clinical drug exerts a novel therapeutic effect on sepsis by suppressing cytokine release, which meant that TFP can enhance accessibility to sepsis treatment through drug reposition.

## Results

### TFP inhibits pro-inflammatory cytokine release in diverse PAMP-stimulated state

**Fig. 1:**
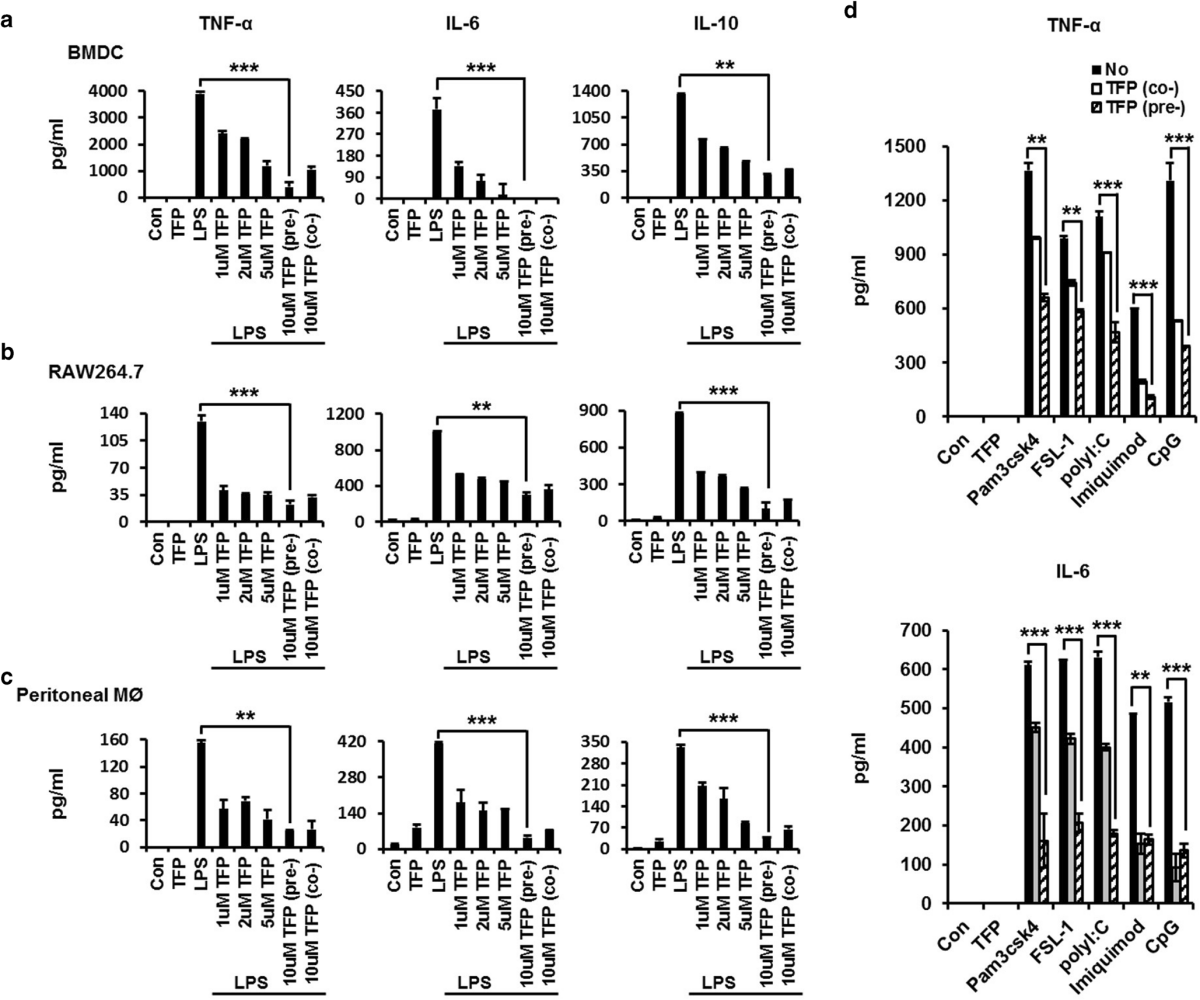
TFP inhibits pro-inflammatory cytokine release in diverse PAMP-stimulated state. To identify the effect of TFP in vitro, **a** bone marrow–derived dendritic cells (1 × 10^6^) from C57BL/6 mice were stimulated with 50 ng/mL LPS at 37 °C for 18 h. The DCs were pretreated with 1, 2, 5, and 10 μM TFP for 30 min or treated concurrently with 10 μM TFP. **b** RAW 264.7 cells (1 × 10^5^) were pretreated with 1, 2, 5, and 10 μM TFP for 30 min before stimulation with 50 ng/mL LPS or treated concurrently with 10 μM TFP at 37 °C for 18 h. **c** Residual macrophages (1 × 10^6^) collected from the abdominal cavity of C57BL/6 mice were treated with the same conditions as above. The control group was nontreated cells, and the LPS-only group served as a positive control. After incubation, supernatants were collected from all groups, and the concentration of the pro-inflammatory cytokines TNF-α, IL-6, and IL-10 were determined using ELISA. Each bar graph represents cytokine level. **d** Bone marrow DCs (1 × 10^6^) were stimulated at 37 °C for 18 h with TLR agonists: 10 μg/mL Pam3CSK4 (TLR1/2), 100 ng/mL FSL-1 (TLR2/6), 50 μg/mL Poly(I:C) (TLR3), 10 μg/mL imiquimod (TLR7), and 50 μg/mL ODN1826 (TLR9). The white square group was treated concurrently and the striped square group pretreated with 10 μM TFP. TNF-α and IL-6 cytokine levels were estimated using ELISA. Significant differences are indicated by *P < 0.05, **P < 0.01, and ***P < 0.001

Upon infection by bacteria and virus, phagocytic cells are generally activated by the LPS, which is known as the main component of the outer membrane of gram-negative bacteria, and then secrete various pro- or anti-inflammatory cytokines and surface molecules [26]. We examined whether TFP prevents protein secretion. DCs were obtained from the bone marrow of C57BL/6 mice incubated with 1, 2, 5, and 10 μM TFP before 30 min or treated simultaneously with 50 ng/mL LPS for 18 h in the presence of TFP. DC activation was determined by measuring LPS-induced release of pro- and anti-inflammatory cytokines using enzyme-linked immunosorbent assay (ELISA). The results showed that the concentrations of cytokines, such as TNF-α, IL-6, and IL-10, in the TFP + LPS group increased compared with those in the control. However, compared to the cells treated with LPS only, the secretion of all pro-inflammatory cytokines significantly decreased in the TFP-treated group in dose-dependent manner (Fig. 1a). Moreover, TFP pretreatment for 0.5 h or 1 h decreased the secretion of the maturation surface markers CD40, CD80, and MHC-I in DCs, as determined by FACS analysis (Supplementary Fig. 1a). The same effects were observed not only in RAW264.7 cells (Fig. 1b) but also in peritoneal residential macrophages (Fig. 1c). In addition, single- or double-strand RNA, DNA, and bacterial lipoprotein released from virus or gram-positive bacteria can stimulate phagocytic cells and LPS [12, 27]. Thus, we investigated whether TFP inhibits DC activation by other pathogen-associated molecular patterns (PAMPs). Dendritic cells were pretreated 1, 2, 5, and 10 μM TFP for 30 min or treated concurrently with 10 μM TFP and then stimulated with 10 μg/mL Pam3CSK4 (lipopeptide), 100 ng/mL FSL-1 (lipoprotein), 50 μg/mL poly(I:C) (dsRNA), 10 μg/mL imiquimod (ssRNA), and 50 μg/mL ODN1826 (DNA) for 18 h in the presence of TFP. We then determined the levels of TNF-α and IL-6, as DC activation markers in the supernatant, using ELISA. As shown in Fig. 1d, TFP suppressed the secretion of pro-inflammatory cytokine by diverse pathogenic molecules. These results suggested that TFP inhibited LPS-induced activation by suppressing pro-inflammatory cytokine secretion in diverse PAMP-stimulated state.

### TFP administration enhances survival of LPS-induced endotoxemia and CCI-induced sepsis models

**Fig. 2:**
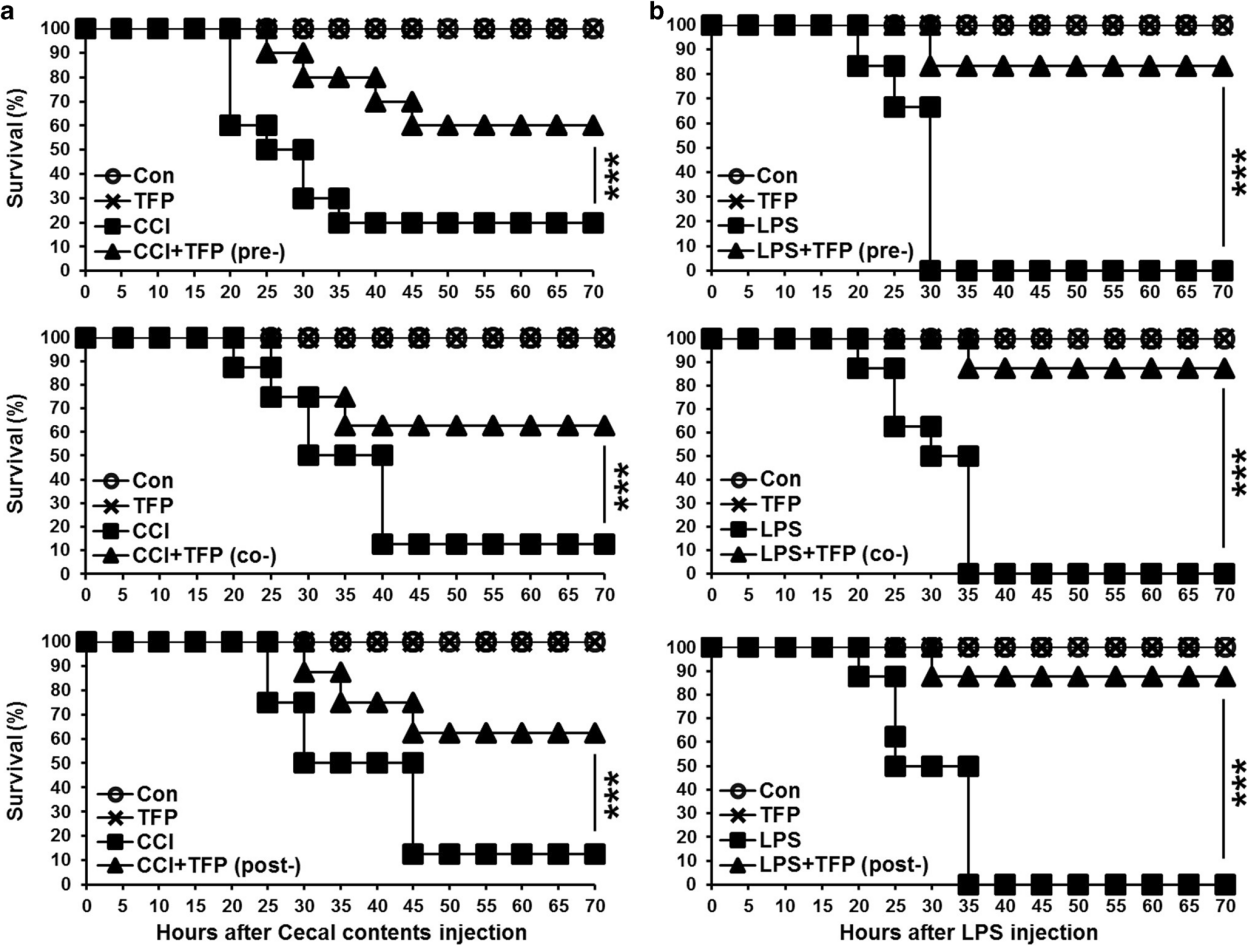
TFP administration enhances survival in LPS-induced endotoxemia and cecal content–injected sepsis models. To mimic conditions of sepsis, **a** 20 mg of cecal contents were injected to C57BL/6 mice, which were equally administered 5 mg/kg TFP 30 min before or after into the peritoneal cavity. A group in the middle line means a concurrent treatment with TFP. To establish endotoxemia model, **b** C57BL/6 mice were injected intraperitoneally with a lethal dose of LPS at 100 mg/kg; 5 mg/kg TFP were administrated intraperitoneally, and then the animals were monitored for 70 h. The line graphs of **a** and **b** describe survival of each group. All group consisted of six mice, and the experiment was conducted in triplicate. ***Significant differences (*P* < 0.001) from the data of the LPS-only group. Results are representatives of three independent experiments

To evaluate the therapeutic efficacy of TFP in a polymicrobial septic condition, a CCI-induced sepsis model was generated [28]. C57BL/6 mice were intraperitoneally injected with 20 mg/mouse of collected cecal contents. Before that, each mouse was equally injected with 5 mg/kg TFP for 30 min into the peritoneal cavity group (Fig. 2a). The TFP-administered group showed a significant 40% increase in survival rate compared with the CCI-only group. In addition, the same therapeutic effect was also observed when TFP was simultaneously and post-treated (Fig. 2a). We also confirmed the protective effect of TFP in the LPS-induced endotoxemia model as follows. We intraperitoneally injected C57BL/6 mice with a lethal dose of LPS (100 mg/kg). Each mouse was intraperitoneally injected with 5 mg/kg TFP 30 min before, after, or at the same time. Similarly, the TFP-treated group exhibited a significant 80% increase in survival rate compared to the LPS-only group (Fig. 2b). This implied that TFP exerted a therapeutic effect in the LPS-induced endotoxemia and CCI-injected sepsis models.

### TFP impedes pathogen infection-induced cytokine secretion in vivo

**Fig. 3:**
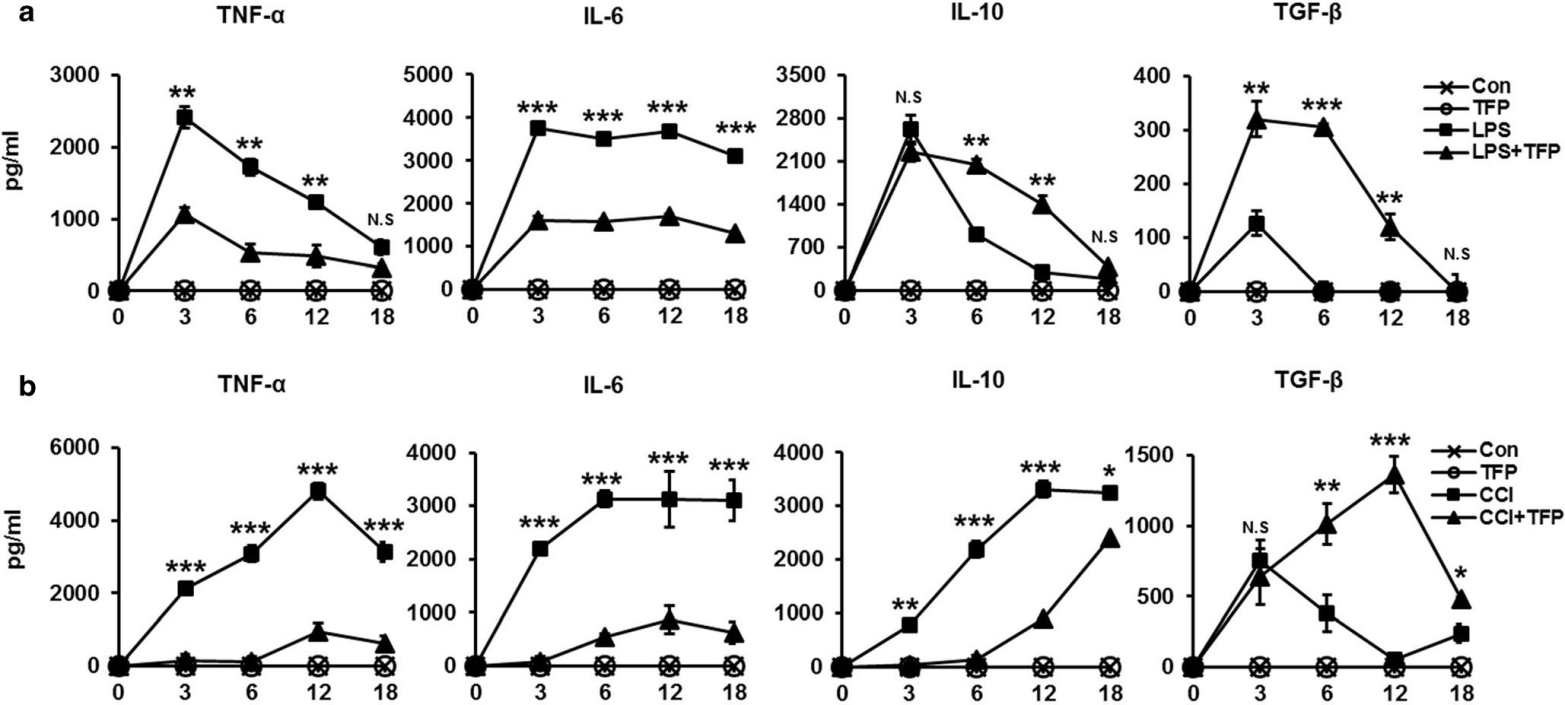
TFP impede cytokine secretion induced by pathogen infection in vivo. C57BL/6 mice were pretreated with 5 mg/kg TFP for 30 min and treated with **a** 20 mg/mouse cecal content injection or **b** 100 mg/kg LPS. To certain pro-inflammatory and anti-inflammatory cytokine levels in the serum, whole blood was collected and centrifuged at 13,000 rpm for 15 min. Supernatants were extracted at 3, 6, 12, and 18 h after LPS treatment. Serum concentrations of pro-inflammatory cytokines, TNF-α, IL-6, and IL-10, and the anti-inflammatory cytokine TGF-β were estimated using ELISA. Con group indicates healthy mice, and six mice were used in the experiment. Each cytokine concentration is illustrated as a line plot. Significant differences at *P < 0.05, **P < 0.01, and ***P < 0.001 at each time point between the LPS-only and TFP + LPS groups. The *t* test was used for statistical analysis

Pathogenic organisms spread throughout the body through the blood stream. In response to multiple infectious agents, phagocytic cells are activated and they release excessive cytokines all over the host’s body. This leads to a whole-body inflammatory response [12]. Thus, we examined whether TFP, which exhibited an inhibitory effect on cytokine release, is effective against LPS-induced endotoxemia and CCI-induced sepsis. To evaluate pro- and anti-inflammatory cytokine production in vivo, we generated CCI-induced sepsis and LPS-induced endotoxemia models. After 30 min of TFP treatment, LPS or cecal contents were injected intraperitoneally and serum was isolated at each time point. Changes in the serum levels of the pro- and anti-inflammatory cytokines TNF-α, IL-6, IL-10, and TGF-β were determined following TFP administration in mice treated with LPS. As shown in Fig. 3a, LPS injection increased the serum levels of TNF-α, IL-6, IL-10, and TGF-β, which reached peaks within 3 h after LPS injection and then slowly decreased. Notably, the TFP-treated group showed a remarkable decrease in TNF-α and IL-6 levels at each experimental time point. On the contrary, the concentrations of IL-10 and TGF-β, which are known to suppress inflammation, decreased compared LPS-only group to regulate immoderate inflammatory state as an adaptive immune response. Additionally, we confirmed a similar result in a CCI-induced sepsis model. CCI led to increased serum levels of TNF-α, IL-6, and IL-10, which peaked within 3 or 6 h after CCI. All TFP pretreatment groups appeared to have reduced emission of pro-inflammatory cytokines (Fig. 3b). This indicated that TFP attenuated pro-inflammatory cytokine secretion, thereby increasing survival rate in models of LPS-induced endotoxemia and CCI-induced sepsis.

### TFP reduces organ dysfunction and tissue damage caused by cytokine storm

**Fig. 4:**
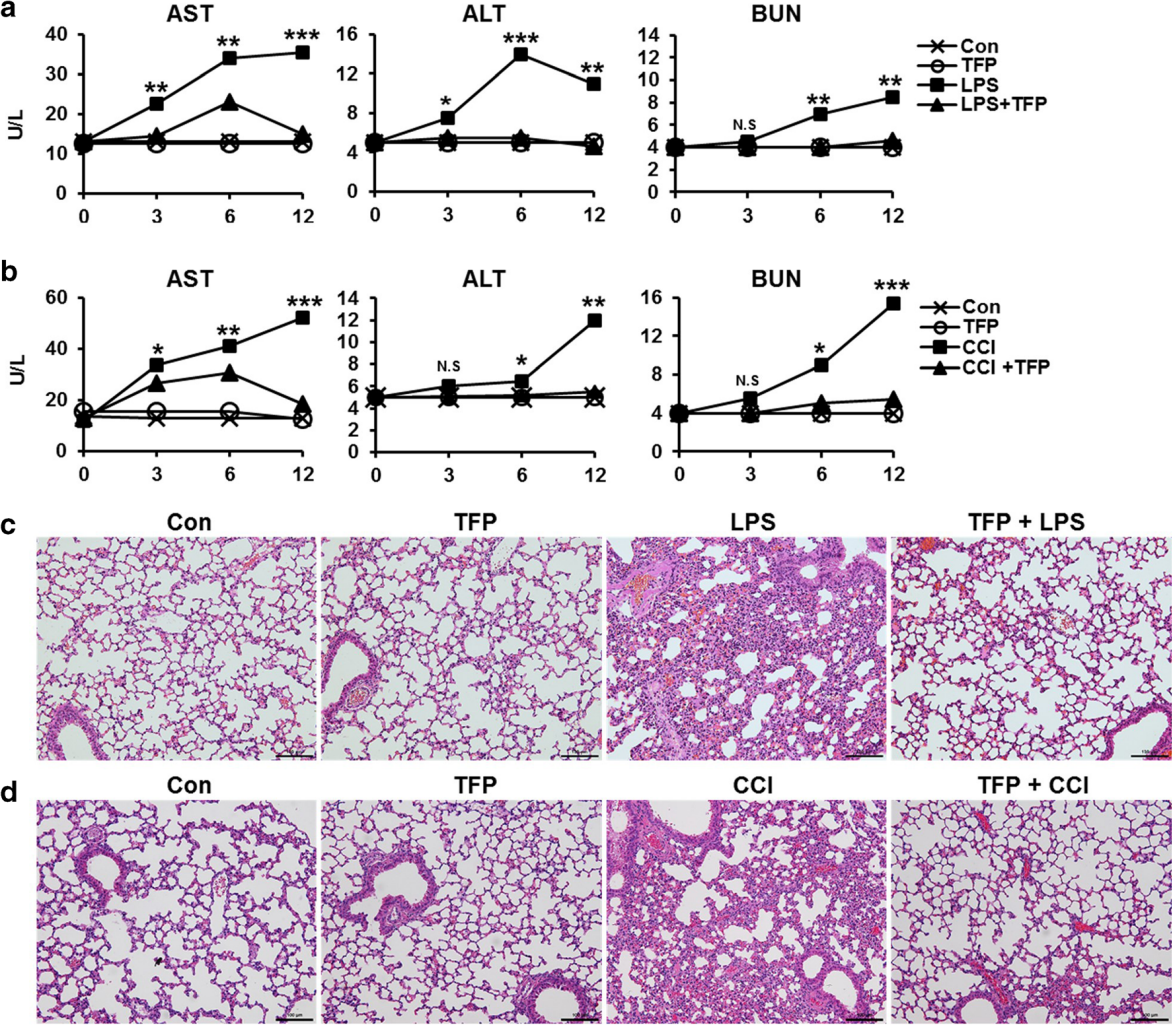
TFP reduces organ dysfunction and tissue damage result in cytokine storm. C57BL/6 mice were pretreated with 5 mg/kg TFP for 30 min by intraperitoneal injection, and then treated with **a**, **c** 100 mg/kg LPS or **b**, **d** 20 mg/mouse cecal content injection (CCI). At 18 h after the treatment, whole blood was collected and serum was extracted. Concentrations of AST, ALT, and BUN enzymes in the serum samples were measured and the results are represented by bar graphs. “No treatment” group at 0 h was used as a control. Data represent three independent experiments. Significant differences at *P < 0.05, **P < 0.01, and ***P < 0.001 at each time point between the LPS-only and TFP + LPS groups. The *t* test was used for statistical analysis. To discern tissue damage and PMN infiltration by LPS and CCI, **c**, **d** lungs were collected, washed with 1× PBS, and fixed with 4% paraformaldehyde at 18 h after the treatment. Paraffin lung sections were stained with hematoxylin and eosin. Representative images of lung sections from each group were captured with a digital camera (Nikon DS-Ri) coupled with a Nikon Eclipse Ni microscope under × 20 magnification. Scale bar, 100 μm

Because dysregulation of inflammatory cytokine production initiates cytokine storm, which leads to tissue damage and multiorgan dysfunction, we hypothesized that tissue damage and organ dysfunction will be weakened because of decreased serum cytokine level following TFP treatment. To assess the extent of tissue damage, we examined the effect of TFP administration on LPS- or cecal content–induced organ damage by measuring serum concentrations of aspartate transaminase (AST), alanine transaminase (ALT), and blood urea nitrogen (BUN) (Fig. 4a, b). Augmentation of these enzymes is known to be positively correlated with liver damage, hepatotoxicity, and kidney dysfunctions. As shown in Fig. 4a, serum levels of AST, ALT, and BUN were gradually increased by LPS injection compared with the non-LPS-treated group. However, their concentrations at the same time point were significantly reduced when TFP was administered before LPS. Equally, we identified the same effect using a CCI-induced model. The concentrations of AST and ALT, as indicators of liver function, were increased following CCI, but TFP treatment downregulated these enzymes. Furthermore, blood BUN level had rapidly been increasing since the 6-h time point. However, TFP-pretreated group showed normal level as control group (Fig. 4b). These results suggested that TFP treatment may attenuate organ damage in LPS-induced endotoxemia or CCI-induced sepsis. Accordingly, cellular infiltration of polymorphonuclear (PMN) leukocytes causes direct tissue damage [29]. To assess the potential reduction of tissue damage by TFP, we examined PMN infiltration in the lungs following LPS or CCI treatment. Obtrusive lung PMN infiltration was observed following LPS- or CCI-only treatment, whereas TFP preadministration relatively reduced PMN infiltration (Fig. 4c, d). This result implied that TFP exerted a protective effect against tissue or organ damage in animal models.

### TFP influences cytokine secretion independently of the signaling and transcriptional level of MAPKs following LPS stimulation

**Fig. 5:**
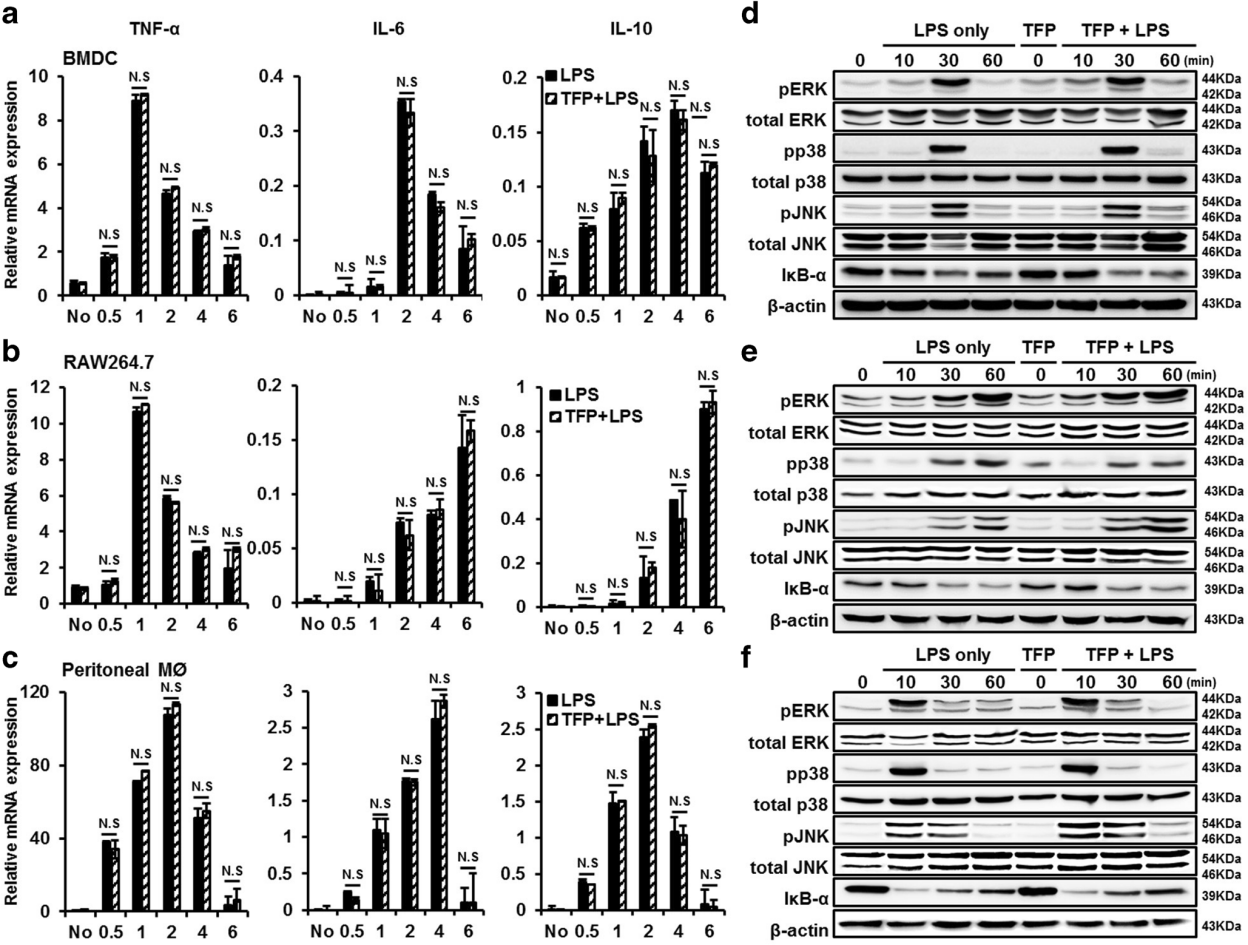
TFP influences cytokine secretion independently of the MAPK signaling and cytokine transcription following LPS stimulus. Bone marrow–derived dendritic cells (DCs) (1 × 10^6^) from C57BL/6 mice (**a**), RAW264.7 cells (**b**), and peritoneal residential macrophages (**c**) were stimulated with 50 ng/mL LPS at 37 °C for 0.5, 1, 2, 4, and 6 h. Before that, the cells were pretreated with 10 μM TFP for 30 min. The transcription level of the TNF-α, IL-6, and IL-10 genes in treated cells were determined using real-time PCR according to the TRIzol protocol. Reference values indicate GAPDH transcriptional level. To measure the levels of MAPK proteins after LPS stimulation. Bone marrow DCs (4 × 10^6^) (**d**), RAW264.7 cells (**e**), and peritoneal residential macrophages (**f**) were treated with 10 μM TFP for 30 min, and then treated with LPS at 50 ng/mL at 37 °C for the indicated time (10, 30, or 60 min). Cell lysates were collected after the indicated time point, and various TLR4 signaling MAPK proteins, MAPKs (ERK, p38, and JNK), IkB-α, and phosphorylated MAPKs (p-ERK, p-p38, and p-JNK) were detected by western blotting. β-Actin was used as a loading control. N.S indicates insignificant difference from the date of the LPS-only group. Similar results were obtained in three separate experiments

We identified that TFP treatment suppressed cytokine secretion induced by various stimulators in vitro and in vivo. Next, we examined the mechanism of TFP at intracellular level. Preferentially, we parallelized the correlation between cytokine concentration at the protein and transcriptional mRNA levels. To estimate transcriptional mRNA levels of cytokines, DCs were obtained from the bone marrow of C57BL/6 mice and incubated with 10 μM TFP for 30 min and then stimulated with 50 ng/mL LPS for 0.5, 1, 2, 4, and 6 h in the presence of TFP. As shown in Fig. 5a, TNF-α, IL-6, and IL-10 mRNA levels were increased by LPS stimulation. Interestingly, however, we were unable to observe a decline in cytokine mRNA level in the TFP treatment group compared to the LPS-only group. The same effects were observed not only in RAW264.7 cells (Fig. 5b) but also in peritoneal residential macrophages (Fig. 5c). These results connoted that TFP did not alter the regulation of the mRNA transcription of cytokines and also did not exclusively alter the secretion of cytokines in LPS-simulated DCs, RAW 264.7, and peritoneal residual macrophages. Next, we examined whether TFP interrupts the downstream signaling of TLR4, a receptor of LPS. LPS binds to the identical receptor complex (TLR4/MD2) and induce TLR4-mediated MAPK phosphorylation to produce pro-inflammatory cytokines [30, 31]. To confirm the relevance of TLR4 signaling in the attenuation of cytokine production by TFP, the levels of various signaling proteins in the TLR4 pathway were assessed by western blotting. DCs were cultured in the presence of 10 μM TFP and stimulated with 50 ng/mL LPS. Each signaling protein was probed with the designated antibodies. As shown in Fig. 5d, high level of phosphorylated ERK, p38, and JNK were detected in DCs within 30 min of LPS-only treatment. Similar to the LPS-only group, the TFP treatment group showed no change in LPS-induced phosphorylation of these proteins in DCs. Furthermore, TFP treatment undoubtedly represented the same degradation of IkB-α, indicating a suppression of NF-kB activation compared with the LPS-only treatment. The same effects were obtained not only in RAW264.7 cells (Fig. 5e) but also in peritoneal residential macrophages (Fig. 5f). These results supported that TFP inhibited pro-inflammatory cytokine production by suppressing cytokine secretion without altering the signaling and transcriptional level of MAPKs.

### Effect of TFP as a W7-like calcium/calmodulin inhibitor

**Fig. 6:**
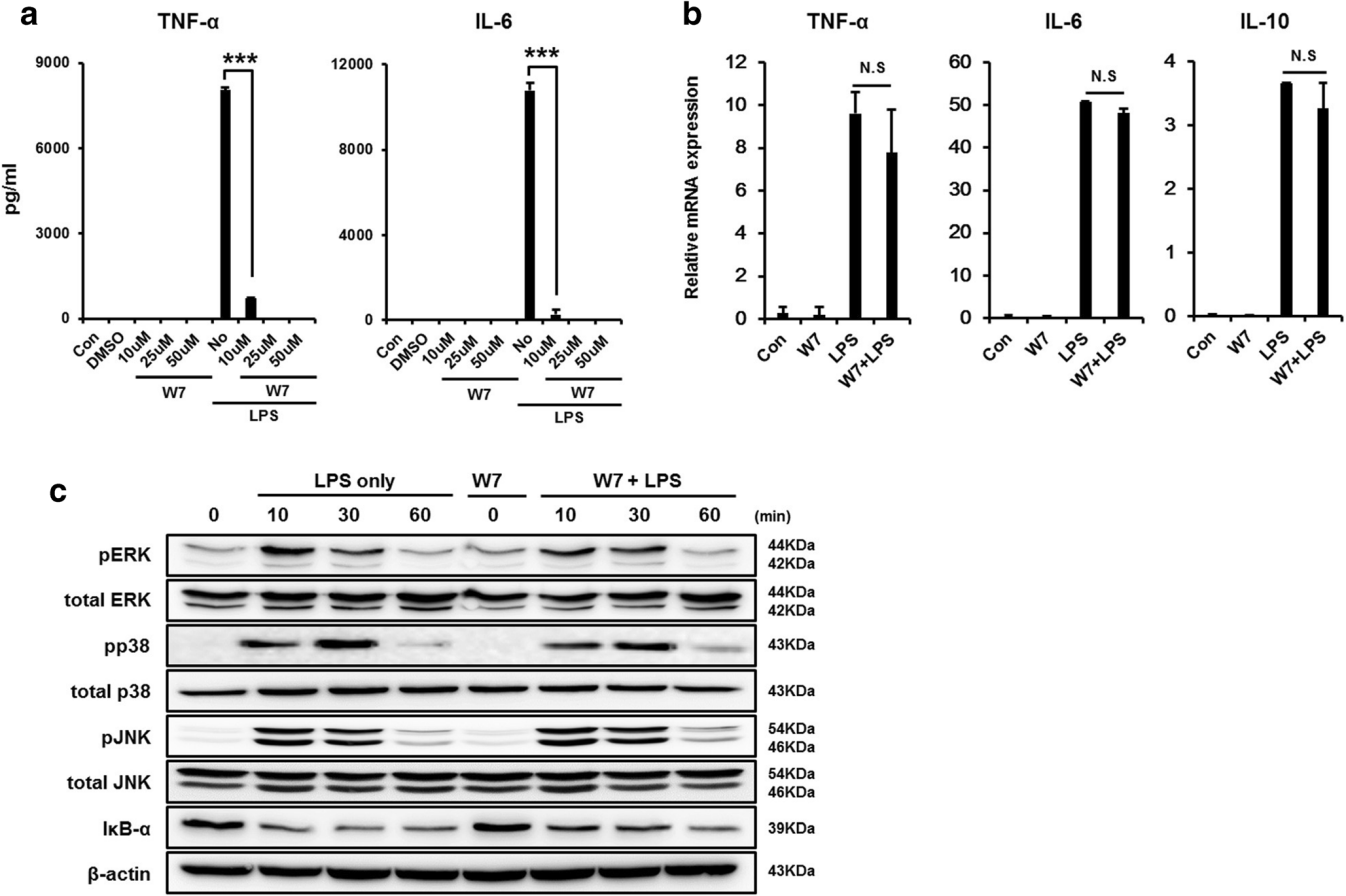
Effect W7-like effect of TFP as a calcium/calmodulin inhibitor. To identify the effect of W7 in vitro, **a** 1 × 10^6^ bone marrow dendritic cells (DCs) from C57BL/6 mice were stimulated with 50 ng/mL LPS at 37 °C for 18 h. DCs were pretreated with 10 μM W7 for 30 min. After incubation, supernatants were collected and the concentrations of pro-inflammatory cytokines, such as TNF-α and IL-6, were measured using ELISA. Each bar graph represents cytokine levels. ***Significant differences (*P* < 0.001) compared with the LPS-only group. To measure the transcriptional levels of cytokines following W7 treatment, **b** 1 × 10^6^ bone marrow DCs purified from C57BL/6 mice were stimulated with 50 ng/mL LPS at 37 °C for 4 h. DCs were pretreated with 10 μM W7 for 30 min. The transcription level of the TNF-α, IL-6, and IL-10 genes were determined using real-time PCR according to the TRIzol protocol. GAPDH transcriptional level was used as a reference value. N.S indicates insignificant differences from the data of the LPS-only group. **c** Bone marrow DCs (4 × 10^6^) pretreated with 10 μM W7 for 30 min, and treated with LPS at 50 ng/mL at 37 °C for the indicated time (10, 30, or 60 min). Cell lysates were collected after the indicated time point and various MAPK proteins, MAPKs (ERK, p38, and JNK), IkB-α, and phosphorylated MAPKs (p-ERK, p-p38, and p-JNK), were detected by western blotting. β-Actin was used as a loading control. N.S indicates insignificant differences from the data of the LPS-only group

TFP was also known to function as a calmodulin inhibitor [32], which prevents calcium from binding to CaM and is involved in the activity of various target proteins [33], such as kinase and phosphatases, to regulate cellular secretion. We verified that W7, a common calmodulin inhibitor [34], has the same inhibitory effect on cytokine secretion as that of TFP. As shown in Fig. 6a, DCs were incubated with 10, 25, and 50 μM W7 for 30 min and then stimulated with 50 ng/mL LPS for 18 h in the presence of W7. The results showed that LPS stimulation increased the concentrations of TNF-α and IL-6. However, as we expected, W7 significantly decreased each cytokine secretion at 10 μM or higher. As well as DC activation, we characterized by measuring LPS-induced DC maturation using FACS analysis. W7 treatment downregulated the secretion of the maturation surface marker CD40 in their group pretreated with W7 for 0.5 h compared to the LPS-only group in a concentration-dependent manner (Supplementary Fig. 2a). W7-pretreated group did not show difference in TNF-α, IL-6, and IL-10 transcriptional levels compared to those in the LPS-only group (Fig. 6b). In addition, we confirmed the relevance of TLR4 signaling pathway in the attenuate of cytokine production by W7. TLR4 downstream signaling proteins were discerned by western blotting. As shown in Fig. 6c, similar to the phosphorylation of ERK, p38, and JNK were detected in the LPS-only group; however, W7 treatment group had the same effect on LPS-induced phosphorylation in DCs. This indicated that W7, as a calmodulin inhibitor, exerted the same inhibitory effect on cytokine secretion as that of TFP. In addition, we have identified the same effects on TFP that increase of intracellular calcium concentration induced by the LPS stimulus is inhibited by the calmodulin inhibitor, W7 (Supplementary Fig. 4a–d) [35, 36]. In conclusion, we concluded that TFP was involved in the regulation of cytokine secretion with the same mechanism as that of W7, as a calmodulin inhibitor.

## Discussion

TFP is an antipsychotic drug mainly used to treat schizophrenia patients by suppressing dopamine secretion. However, another therapeutic potential of TFP has been reported. TFP suppressed the growth of tumor and brain metastasis by inducing G0/G1 cell cycle arrest of triple-negative breast cancer (TNBC) without causing detectable side effects in vivo [37]. TFP, a novel autophagy inhibitor, increases radiosensitivity in glioblastoma by impairing homologous recombination [38]. Schubart et al. showed that TFP inhibits insulin secretion from transplantable hamster insulinoma cells. In accordance with this result, this study showed the ability of TFP to inhibit cytokine secretion by stimulated and activated phagocytic cells. We confirmed that TFP reduced the secretion of the pro- and anti-inflammatory cytokines TNF-α, IL-6, and IL-10 in LPS-stimulated DCs (Fig. 1a–c). Cytokine production, however, is not solely induced by LPS, which is known as the main component of the cell wall of gram-negative bacteria. Additionally, we found that TFP inhibited cytokine production induced by the single- or double-strand RNA, DNA, and lipoprotein released from virus or gram-positive bacteria (Fig. 1d). We postulated that the protective effect of TFP in models of LPS-induced endotoxemia and CCI-induced sepsis was significant and without any particular side effect (Fig. 2a, b). Our findings further showed the role of TFP as an inhibitor of systemic inflammation through blockage of cytokine secretion to the serum (Fig. 3). Thus, we have shown, both in vitro and in vivo, that TFP suppressed the levels of the pro-inflammatory cytokines TNF-α and IL-6 in a state of abnormal infection. Importantly, because multiple organ dysfunction caused by sepsis is critical for survival, we also identified that TFP treatment improved tissue damage and liver function, as well as reduce hepatotoxicity and kidney dysfunctions in sepsis models by maintaining normal AST, ALT, and BUN levels (Fig. 4a, b). Additionally, because septic shock leads to multiple organ dysfunctions, we confirmed that lung failure recovered to a normal condition, preventing infiltration of lung PMN (Fig. 4c, d). Moreover, we should consider the potential correlation between TFP and the transcriptional levels of these proteins. As shown in Fig. 5a–c, TFP appeared to have no correlation with the transcriptional levels of these proteins. TFP did not alter the regulation of the mRNA levels of the cytokines and did not exclusively engage in the secretion of cytokines in LPS-simulated DCs, RAW 264.7 cells, and peritoneal residual macrophages. Furthermore, LPS binds to the identical receptor complex (TLR4/MD2) and imposes TLR4-mediated phosphorylation of MAPKs to produce pro-inflammatory cytokines. We found that TFP did not interrupt the downstream signaling of TLR4, which is known as a receptor of LPS (Fig. 5d–f). Our additional results supported these findings in that TFP inhibited pro-inflammatory cytokine production by suppressing cytokine secretion without altering the signaling and transcriptional levels of MAPKs. Blockage of cytokine secretion, however, cannot fully explain the mechanism of TFP. Previous studies reported that dysregulated Ca^2+^ handling is prevalent to organ dysfunction and tissue damage in sepsis. Inhibition of calcium/calmodulin-dependent protein kinase Iα increased survival rate by reducing systemic concentrations of IL-10, IL-6, TNF-α, and HMGB1 in a CLP model of sepsis [39]. Particularly, the current study showed that calmodulin antagonists abrogated activated immune cell–mediated cytokine secretion in DCs. In accordance with these results, we expected TFP to have an equivalent effect with that of W7, a calmodulin inhibitor. As shown in Fig. 6a, W7 pretreatment significantly decreased the secretion of each cytokine irrespective of the transcriptional level and downstream signaling of TLR4. Our results supported that TFP was involved in the regulation of cytokine secretion with the same mechanism as the calmodulin inhibitor W7.

In summary, this study reported that TFP, as a calmodulin inhibitor, inhibited cytokine secretion. This is a new approach to treat sepsis. The discovery of traditional drugs is inefficient and too costly. Recently, reposition of FDA-approved therapeutics for other diseases is regarded as a rapid, alternative approach to develop drugs [40]. Therefore, our findings brought a new approach to treat sepsis for investigation in future clinical trials. The exact mechanism of TFP in inhibiting cytokine secretion in sepsis remains to be elucidated in future studies.

## Experimental procedures

### Mice

Six- to eight-week-old female C57BL/6 mice, weighing 16–18 g, were purchased from Orient Bio, Inc. All animal procedures were approved by and performed according to guidelines of the Institutional Animal Care and Use Committee (IACUC) of Konkuk University. To study survival rate, humane endpoints were used to minimize suffering. In case clinical signs of the moribund state were recognized, the animals were euthanized by CO_2_ euthanasia (PMID: 14676679). The animals were placed in a CO_2_ chamber, and a low flow CO_2_ gas was administered. CO_2_ gas (100%) was administered for another 5 min after the animals lost consciousness.

### Cells

To isolate DCs, monocytes were isolated from the bone marrow of C57BL/6 mice, which were then cultured in RPMI 1640 medium (Biowest, USA) supplemented with 10% fetal bovine serum, 50 U/mL penicillin/streptomycin, 2 mmol/L L-glutamine, 1 mmol/L sodium pyruvate, 2 mmol/L nonessential amino acids, 10 ng/mL granulocyte-macrophage colony-stimulating factor (GM-CSF) (Peprotech), and 5 ng/mL IL4 (Peprotech) at 37 °C and 5% CO_2_. The monocytes were incubated for 6 days before use in the experiment. The murine macrophage RAW 264.7 cell line was purchased from ATCC and cultured in DMEM supplemented with 10% fetal bovine serum and 50 U/mL penicillin/streptomycin. The cells were cultured at 37 °C in a humidified atmosphere cultivator with 5% CO_2._ To isolate peritoneal resident macrophages, peritoneal cells were collected by washing the peritoneal cavity with 10 mL of 1× phosphate-buffered saline (PBS) and then centrifuged. Pellets were resuspended with RBC lysis buffer and washed twice using 1× PBS. The acquired cells were sorted according to the MACS CD11b MicroBeads protocol (Miltenyi Biotec, Germany) and cultured in DMEM/F12 medium supplemented with 10% fetal bovine serum and 50 U/mL penicillin/streptomycin.

### Reagents

The following reagents were used in this study: TFP (Sigma-Aldrich, USA), W-7 (calmodulin inhibitor) (Enzo Life Sciences, USA), in vitro LPS (from *E. coli* O111:B4; InvivoGen, USA), in vivo LPS (*E. coli* serotype O127:B8; Sigma Aldrich), CD11b+ cell isolation kit and LS columns (Miltenyi Biotec), RPMI 1640, DMEM, DMEM/F12, and fetal bovine serum (FBS) (Biowest). Pam3CSK4, FSL-1, poly(I:C), imiquimod, and ODN1826 (InvivoGen, USA).

### Flow cytometry

DCs were stained with a fluorescein isothiocyanate (FITC)-conjugated CD11c DC surface antibody, as well as with phycoerythrin (PE)-conjugated CD40, CD80, and MHC-I (Biolegend, USA) DC maturation antibodies. The cells were analyzed using a FACS Calibur cytometer equipped with the BD CELL Quest Pro software.

### CCI-induced sepsis model

A mouse model of CCI-induced sepsis was generated as previously described (23841524). The mice were euthanized by CO_2_ inhalation, cecectomy was performed, and cecal contents were extracted with a cotton swab into a petri dish. PBS was added to a final concentration of 20 mg/mL, which was then minced using ground glass. Cecal contents were passed through a 100-μm cell strainer to allow a smooth injection. The mice were then intraperitoneally injected with 1 mL of homogenized cecal contents. Each mouse was intraperitoneally injected with 5 mg/kg TFP 30 min before CCI.

### LPS-induced endotoxemia model

Mice were intraperitoneally injected with 100 mg/kg of LPS (*E. coli* serotype O127:B8) (Sigma, USA) dissolved in PBS. Each mouse was intraperitoneally injected with 5 mg/kg TFP 30 min before LPS injection.

### ELISA

For the in vitro and in vivo cytokine analyses, the concentrations of the pro-inflammatory cytokines TNF-α and IL-6 and the anti-inflammatory cytokines IL-10 and TGF-β in cell supernatants or the serum were measured by using commercially obtained ELISA kits. The ELISA kits used to measure TNF-α, IL-6, and IL-10 were all purchased from eBioscience, whereas that used to measure TGF-β was purchased from Becton Dickinson. Each assay was carried out according to the instructions provided by the manufacturers.

### Organ damage

In LPS-induced endotoxemia model and CCI-induced sepsis model, the serum levels of AST, ALT, and BUN were measured by the Konkuk University Hospital Automatic Hematology Analyzer.

### PMN infiltration

Lungs were perfused with 4% paraformaldehyde immediately after being isolated from mice and maintained at 4 °C for 18 h. Fixed lung tissues were washed with distilled water for 2 h to remove paraformaldehyde (PFA). Tissue processing was done using an Auto Leica Tissue Processor 1020 (Leica Biosystems, Germany) which allowed automatic control of tissue infiltration, dehydration, and infiltration under vacuum. Lung tissues were perfused twice in formalin solution for 2 h each. Fixed lungs were then sequentially immersed in 70, 80, 90, and 100% ethanol. After the tissues were immersed in xylene, they were embedded in paraffin and cut into 7-μm-thick sections. Slides were stored for 18 h at 65 °C for deparaffinization. Tissues were hydrated by alcohol and rinsed with distilled water for 10 min. Tissues were then stained with hematoxylin (Merck, USA) and eosin (Merck). Images were captured with a digital camera (NikonDS-Ri1) coupled with a Nikon Eclipse Nimicroscope under × 20 magnification.

### RT-PCR

To measure the transcription level of cytokines, bone marrow DCs were incubated with LPS in the presence or absence of TFP and W7. After 4 h of LPS treatment, the cells were collected and centrifuged. Pellets were resuspended in 0.2 mL chloroform (Sigma-Aldrich) and 1 mL TRIzol Reagent (Invitrogen, USA). After 3 min of incubation, homogenized cells were centrifuged at 12,000 rpm for 15 min at 4 °C, and the top layer of aqueous phase was collected. RNA was precipitated with isopropanol and washed with 75% ethanol. Acquired RNA was subjected to reverse transcription to synthesize cDNA using PCR. PCR products were analyzed with the Light-Cycler 480 software (Roche) using SYBT Green dye to detect double-stranded DNA.

### Western blotting analysis

Bone marrow DCs were incubated with LPS (50 ng/mL) in the presence or absence of TFP and W7 for 0, 10, 30, or 60 min. Cells were scraped, washed, centrifuged, and resuspended on ice in RIPA protein extraction solution [50 mmol/L Tris-Cl (pH 8.0), 150 mmol/L NaCl, 1 mmol/L phenylmethylsulphonyl fluoride (PMSF), 0.1% sodium dodecyl sulfate (SDS), 1% Nonidet P-40 (NP40), and 0.5 mmol/L EDTA; Elpis Biotech] for 1 h. Protein concentrations were determined by the Bradford protein assay. Proteins of an equal quantity were mixed with the SDS-PAGE loading buffer (250 mmol/L Tris-HCl, pH 6.8, 0.5 mol/L DTT, 10% SDS, 0.5% bromophenol blue, and 50% glycerol), boiled for 10 min, separated by 12% SDS-PAGE and transferred to polyvinylidene difluoride membranes (Roche, Ltd). The membranes were probed with mouse antibodies against JNK, p-JNK, p38, p-p38, ERK, p-ERK, IkB-α (Cell Signaling Technology), and β-actin (Sigma) diluted to 1:1000 in 5% bovine serum albumin and incubated with a goat anti-mouse IgG (Abbiotec) conjugated to horseradish peroxidase secondary antibodies (Enzo Life Sciences). Immunoreactive bands were visualized by enhanced chemiluminescence reaction.

### Cytotoxicity assay

DCs were incubated with TFP and W7 at various concentrations for 18 h. After treatment, the cells were stained with PE-conjugated CD11c DC surface antibody and FITC-conjugated AnnexinV with AnnexinV binding buffers for 15 min, and then analyzed using a FACS Calibur cytometer equipped with the BD CELL Quest Pro software.

### [Ca^2+]^_i_ measurement

BMDCs were incubated with 5 μM Fura-2 AM (intracellular Ca^2+^ level indicator (Thermo Fisher Scientific, Waltham, MA, USA)) for 30 min at room temperature. The incubated cells were then loaded into the patch-clamp chamber and washed in Normal Tyrode solution(143 mM NaCl, 5.4 mM KCl, 0.33 mM NaH_2_PO_4_, 0.5 mM MgCl_2_, 5 mM HEPES, 2 mM CaCl_2_, and 11 mM glucose (pH 7.4 with NaOH)) for 30 min at room temperature. For measurement for Fura-2, excitation and emission were at 340 and 510 nm (Lambda DG-4, Sutter Instrument Company, Navato, CA, USA). Origin 8.0 was used for data analysis.

### Statistical analysis

Data presented in this study were obtained from one representative experiment of the two or three experiments performed. All data were presented as the mean ± standard deviation (SD) of three independent experiments. Individual data points were compared by the Student’s *t* test. Survival of mice was analyzed by the Kaplan–Meier method followed by the log-rank test. Analysis was performed using the SPSS software (version 22.0). Differences between groups were considered significant at *P < 0.05, **P < 0.01, and ***P < 0.001.

## Electronic supplementary material

Supplementary Figure 1 (JPG 305 kb)

Supplementary Figure 2 (JPG 174 kb)

Supplementary Figure 3 (JPG 305 kb)

Supplementary Figure 4 (JPG 337 kb)

ESM 1 (DOCX 14 kb)
