# Supplementary figures and images for: Repositioning of the antipsychotic drug TFP for sepsis treatment
Source: J Mol Med (Berl). 2019 Mar 8;97(5):647–58. doi: 10.1007/s00109-019-01762-4 (PMC6488556; doi:10.1007/s00109-019-01762-4)

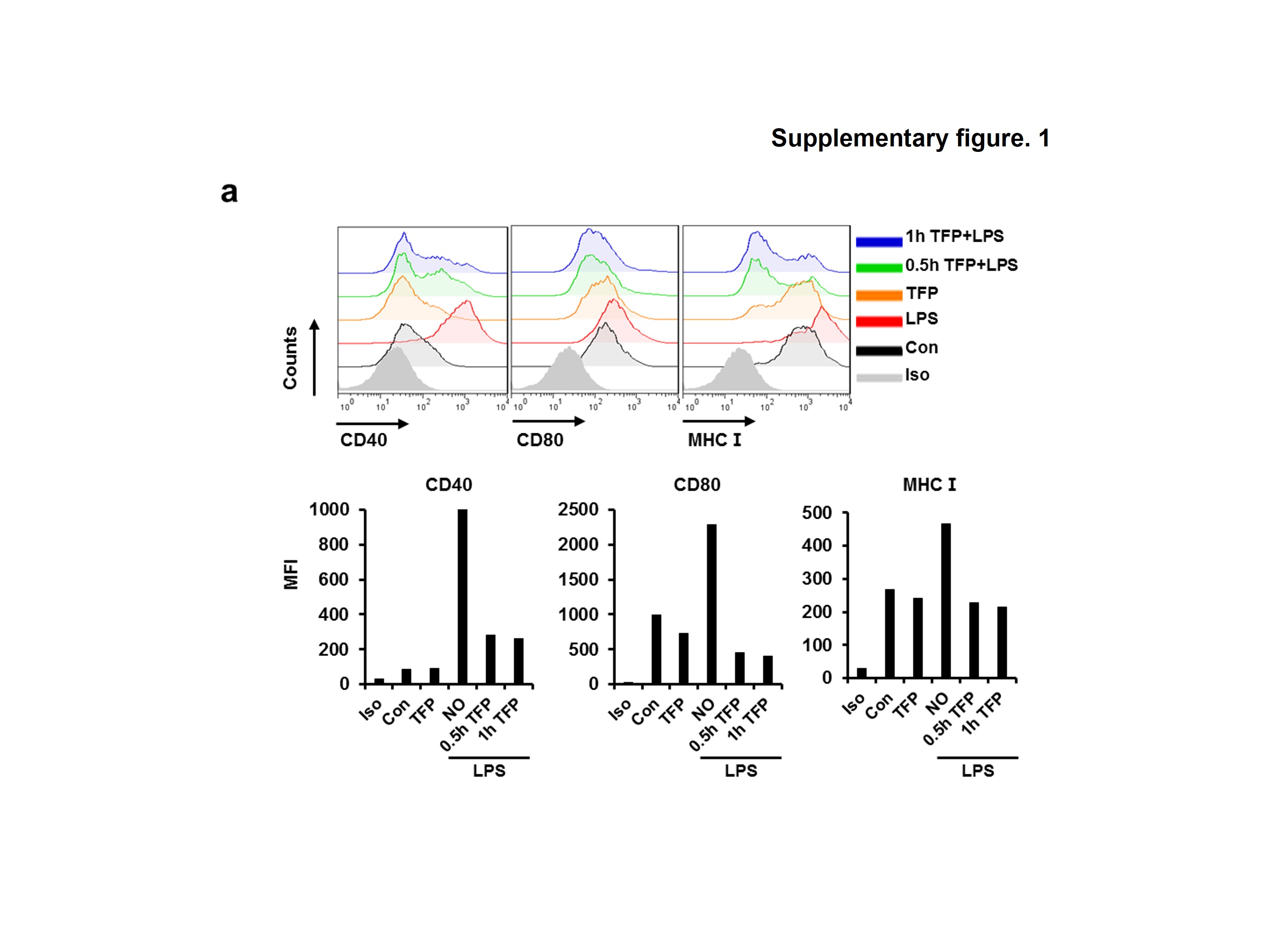

Supplement: Supplementary file 1 — (JPG 305 kb) [file 109_2019_1762_MOESM1_ESM.jpg]

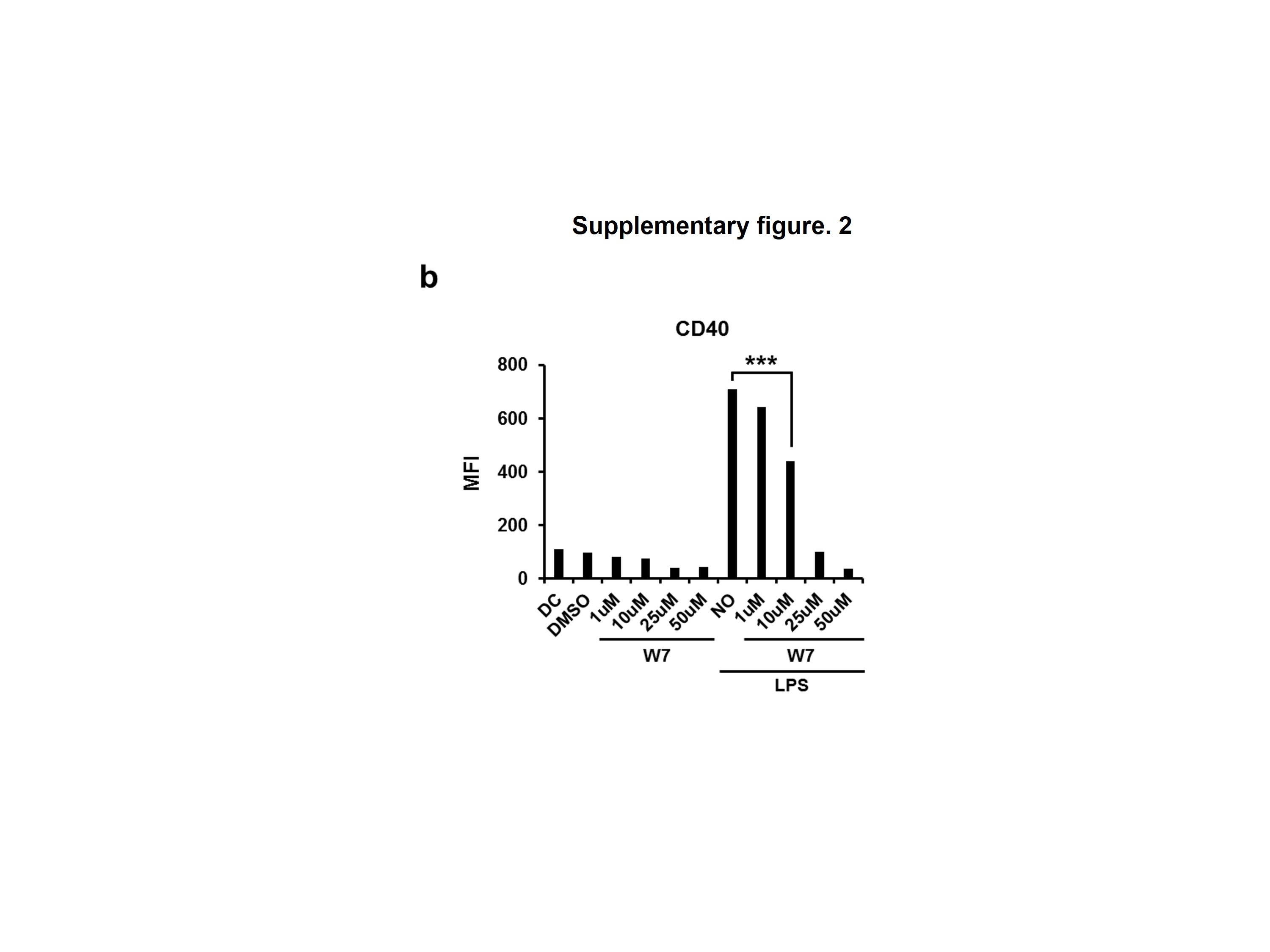

Supplement: Supplementary file 2 — (JPG 174 kb) [file 109_2019_1762_MOESM2_ESM.jpg]

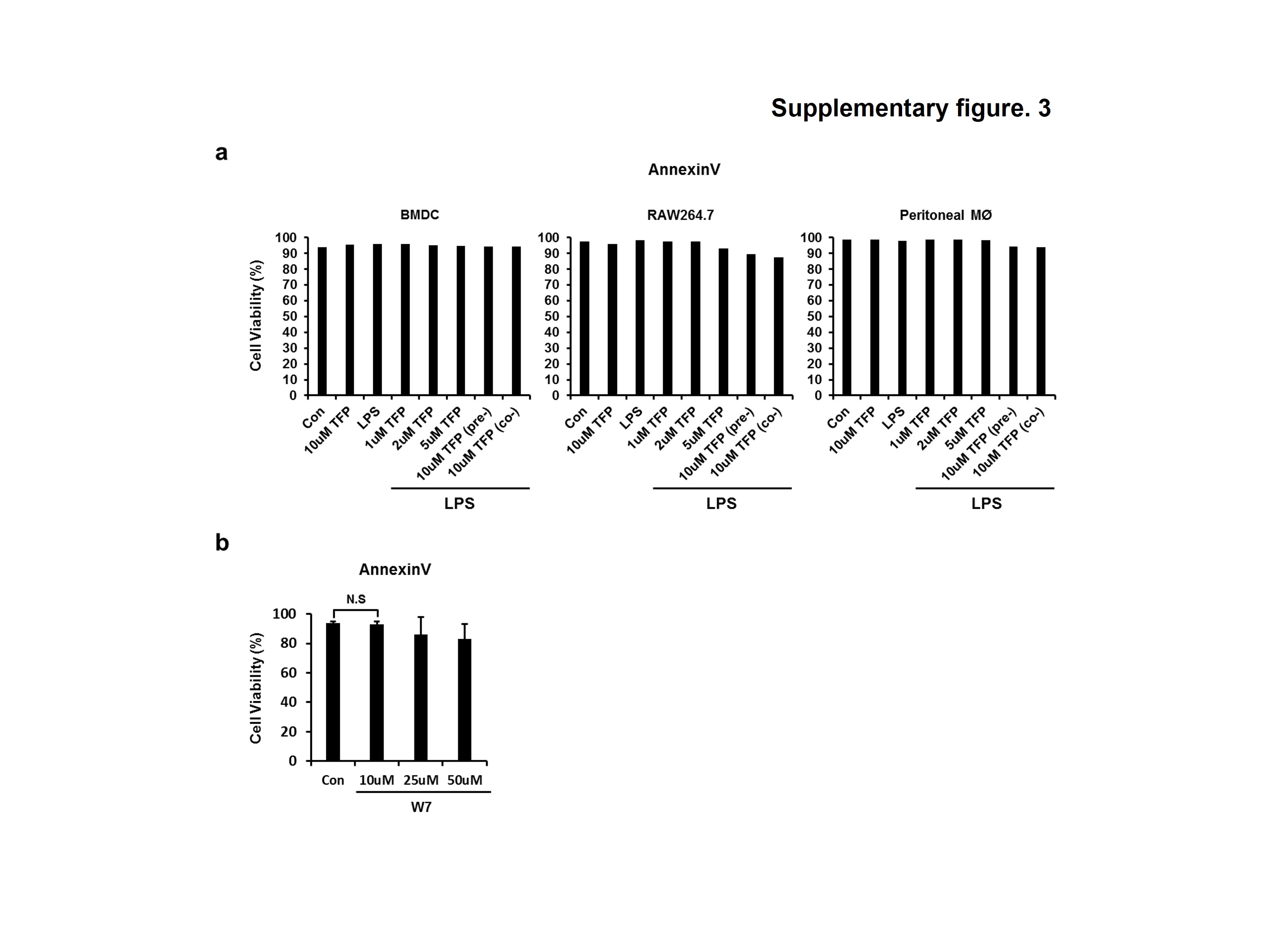

Supplement: Supplementary file 3 — (JPG 305 kb) [file 109_2019_1762_MOESM3_ESM.jpg]

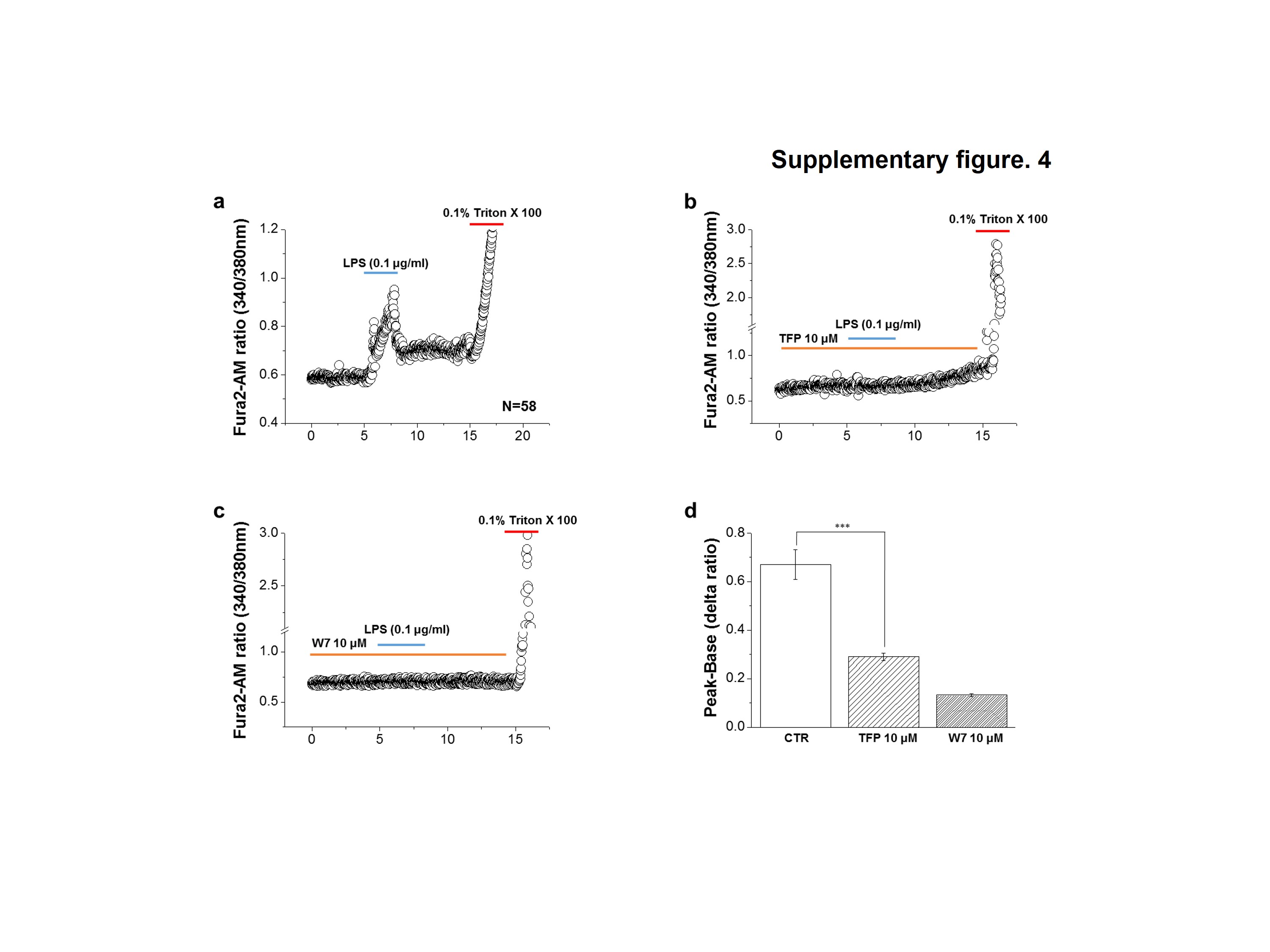

Supplement: Supplementary file 4 — (JPG 337 kb) [file 109_2019_1762_MOESM4_ESM.jpg]
