# Supplementary material for: Repositioning of the antipsychotic drug TFP for sepsis treatment
Source: J Mol Med (Berl). 2019 Mar 8;97(5):647–58. doi: 10.1007/s00109-019-01762-4 (PMC6488556; doi:10.1007/s00109-019-01762-4)
Supplement: Supplementary file 5 — (DOCX 14 kb) [file 109_2019_1762_MOESM5_ESM.docx]

Repositioning of the antipsychotic drug TFP for sepsis treatment

Journal of Molecular Medicine

Jung Hwa Park^1^, Hyun Jin Park^1^, Sung Eun Lee^1^, Young Seob Kim^1^, Gun-Young Jang^1^, Hee Dong Han^1^, In Duk Jung^1^, Kyung Chul Shin^2^, Young Min Bae^2^, Tae Heung Kang^*^ and Yeong-Min Park^*^

^1^ Department of Immunology, School of Medicine, KonKuk University, Chungju 27478, South Korea

^2^ Department of Physiology, School of Medicine, KonKuk University, Chungju 27478, South Korea

* To whom correspondence: Tae Heung Kang, Department of Immunology, School of Medicine, Konkuk University, 268, Chungwondaero, Chungju 27478, South Korea. Tel: 82-2-2049-6089; Fax: 82-2-2049-6192; E-mail: kangiron@kku.ac.kr; Yeong-Min Park, Department of Immunology, School of Medicine, Konkuk University, 268, Chungwondaero, Chungju 27478, South Korea. Tel: 82-2-2049-6158; Fax: 82-70-8280-0378; E-mail: immun3023@kku.ac.kr

**Supplementary Figure**

**Supplementary fig 1. TFP reduces LPS-induced maturation of DCs.**

**(a)** 1 × 10^6^ bone marrow dendritic cells (DCs) from C57BL/6 mice were pre-treated with 10 µM TFP for 0.5 h or 1 h and stimulated with 50 ng/mL LPS at 37℃. After 18 h, the levels of DC maturation markers were estimated. CD40, CD80, and MHC-Ⅰ levels on DCs were measured using flow cytometry analysis. The bar graph illustrates the mean fluorescence intensity (MFI) of each surface marker. LPS treatment was used as a positive control and immature DCs served as a negative control.

**Supplementary fig 2. W7 reduces LPS-induced maturation of DCs.**

**(a)** 1 × 10^6^ bone marrow dendritic cells (DCs) from C57BL/6 mice were pre-treated with 1, 10, 25, or 50 µM W7 for 0.5 h and stimulated with 50 ng/mL LPS at 37℃. After 18 h, the levels of DC maturation markers were estimated. CD40 level on DCs were measured using flow cytometry analysis. The bar graph illustrates the mean fluorescence intensity (MFI) of each surface marker. LPS treatment was used as a positive control and immature DCs served as a negative control.

**Supplementary fig 3. Cytotoxicity of TFP and the calmodulin inhibitor W7.**

To confirm cytotoxicity**, (a)** 1 × 10^6^ bone marrow dendritic cells (DCs) from C57BL/6 mice, RAW264.7 cells and peritoneal residential macrophages were pre-treated with TFP (1, 2, 5 or 10 µM) for 0.5 h or concurrently and then stimulated with 50 ng/mL LPS at 37℃. **(b)** 1 × 10^6^ BMDCs were pre-treated with W7 (10, 25, or 50 µM) for 0.5 h and then stimulated with 50 ng/mL LPS at 37℃. After 18 h, the cells were collected and stained with FITC-conjugated AnnexinV antibodies for 20 min at 4°C. The bar graph illustrates the mean fluorescence intensity (MFI) of AnnexinV-positive cells.

**Supplementary fig 4. Effect of TFP and the calmodulin inhibitor W7 on intracellular Ca^2+^ concentration ([Ca^2+]^ _i_).**

**(a)** Representative trace showing that LPS (0.1µg/ml) increase intracellular [Ca^2+]^. **(b-c)** Representative Ca^2+^ trace showing the effects of pretreatment with 10µM TFP and W7 before 30min. **(d)** Summarized data showing the effects of blockers of intracellular Ca^2+^ on TFP and W7. Y axis represents the F340/F380 ratio value.
